# Implication of molecular vascular smooth muscle cell heterogeneity among arterial beds in arterial calcification

**DOI:** 10.1371/journal.pone.0191976

**Published:** 2018-01-26

**Authors:** Olivier Espitia, Mathias Chatelais, Marja Steenman, Céline Charrier, Blandine Maurel, Steven Georges, Rémi Houlgatte, Franck Verrecchia, Benjamin Ory, François Lamoureux, Dominique Heymann, Yann Gouëffic, Thibaut Quillard

**Affiliations:** 1 INSERM, UMR 1238, Nantes, France; Université de Nantes, Nantes Atlantique Universités, Laboratoire « Sarcome osseux et remodelage des tissus osseux calcifiés », Faculté de Médecine, Nantes, France; 2 CHU Hôtel Dieu, Nantes, France; 3 Institut du Thorax, Inserm UMR1087, Faculté de Médecine, Université de Nantes, Nantes Atlantique Universités, Nantes, France; 4 Inserm U954, Faculty of Medicine, Nancy, France, DRCI, University Hospital of Nancy, Nancy, France; 5 Institut de Cancérologie de l'Ouest, site René Gauducheau, Boulevard Professeur Jacques Monod, Saint-Herblain, France; 6 University of Sheffield, Department of Oncology and Metabolism, INSERM, European Associated Laboratory “Sarcoma Research Unit”, Medical School, Sheffield, United Kingdom; 7 University of Nantes, Faculty of Medicine, Nantes, France; Duke University, UNITED STATES

## Abstract

Vascular calcification is a strong and independent predictive factor for cardiovascular complications and mortality. Our previous work identified important discrepancies in plaque composition and calcification types between carotid and femoral arteries. The objective of this study is to further characterize and understand the heterogeneity in vascular calcification among vascular beds, and to identify molecular mechanisms underlying this process. We established ECLAGEN biocollection that encompasses human atherosclerotic lesions and healthy arteries from different locations (abdominal, thoracic aorta, carotid, femoral, and infrapopliteal arteries) for histological, cell isolation, and transcriptomic analysis. Our results show that lesion composition differs between these locations. Femoral arteries are the most calcified arteries overall. They develop denser calcifications (sheet-like, nodule), and are highly susceptible to osteoid metaplasia. These discrepancies may derive from intrinsic differences between SMCs originating from these locations, as microarray analysis showed specific transcriptomic profiles between primary SMCs isolated from each arterial bed. These molecular differences translated into functional disparities. SMC from femoral arteries showed the highest propensity to mineralize due to an increase in basal TGFβ signaling. Our results suggest that biological heterogeneity of resident vascular cells between arterial beds, reflected by our transcriptomic analysis, is critical in understanding plaque biology and calcification, and may have strong implications in vascular therapeutic approaches.

## Introduction

Vascular calcification is a strong and independent predictive factor for cardiovascular complications and mortality, the leading cause of death worldwide [1]. Accumulating evidence show that calcification in atherosclerosis mechanically affects plaque stability both directly and indirectly. Destabilizing calcification (approx. 5μm) increases mechanical stress within the fibrous cap due to the important mismatch between the stiff microcalcifications and the collagen-rich cap [2–4]. Moreover, advanced and extensive calcification contributes to plaque stability, while increasing arterial stiffness and hypertension, an important risk factor for plaque rupture [5–8].

Despite being exposed to similar risk factors, peripheral arteries develop heterogeneous lesions. Our previous work showed in a population with similar demographic and biological data that carotid arteries develop predominantly lipid-rich lesions and microcalcifications, while femoral arteries develop fibrotic lesions, with extensive calcification and frequent presence of osteoid tissue [9–11].

Historically considered to be a passive process related to aging, it is now recognized as an active process, closely related to bone metabolism due notably to the presence of actual bone structure and numerous typical bone-related molecules and cell types in calcified lesions. Vascular smooth muscle cells (SMC), and pericytes found in advanced lesions with neovascularization, can mineralize directly when cultured in osteogenic milieu, and could directly contribute to plaque calcification [12,13]. Recent work by Hutcheson et al demonstrated that Macrophages and SMC release matrix vesicles and calcifying exosomes in response to inflammation and apoptosis [14–16]. The formation of matrix vesicles initiates the phospho-calcic nucleation and early hydroxyapatite formation, a similar process found in growth plate in bone.

Molecular mechanisms directly regulating the formation of ectopic calcification in vasculature remain poorly understood. Major bone related molecules (OPG, RANKL, BMP, MGP…) have been found in calcified lesions but specific molecular determinants orchestrating plaque mineralization in arterial beds remain poorly understood [17].

To elucidate these mechanisms, and to better understand plaque calcification heterogeneity among vascular beds, we used our ECLAGEN biocollection of human atherosclerotic lesions from different arterial locations (abdominal and thoracic aorta, carotid, femoral, and infrapopliteal arteries).

Our results show that lesion composition differs between these locations. Femoral arteries are the most calcified arteries overall. They develop more advanced calcifications (sheet-like, nodule), and are highly susceptible to osteoid metaplasia (OM). These discrepancies could derive from intrinsic differences between SMCs originating from these locations. SMC from femoral arteries have the highest propensity to mineralize due to an increase in basal transforming growth factor β (TGFβ) signaling.

## Material and methods

### Patients and biological samples

ECLAGEN biocollection included two main groups of patients. First, atherosclerotic diseased arteries were harvested from patients undergoing arterial open surgery for at least one arterial location: carotid artery (CA), thoracic (TA) and abdominal aorta (AA), and common femoral (FA) and infrapopliteal arteries (PA). Non-atheromatous, healthy arteries were harvested from organ donors. In this case, up to five arterial territories, as previously described, were collected from the same donor. For all patients, demographic data, patient medical history and blood samples were collected. Altogether, ECLAGEN includes 244 patients (217 atherosclerotic patients and 27 organ donors for healthy arteries), recruited in Nantes from october 2011 to december 2015 (Table 1).

**Table 1:** 

	AA (n = 46)	CA (n = 87)	FA (n = 53)	PA (n = 25)	Total (n = 211)	
Smoking	22 (48%)	20 (23%)	22 (42%)	2 (8%)	66 (31.3%)	*<0*,*001*
Hypertension	22 (48%)	65 (75%)	38 (72%)	23 (92%)	148 (70.1%)	*<0*,*001*
Diabetes	5 (11%)	25 (29%)	23 (43%)	18 (72%)	71 (33.6%)	*<0*,*001*
Dyslipidemia	31 (67%)	68 (78%)	30 (57%)	12 (48%)	141 (66.8%)	*0*,*009*
Gender (M)	40 (87%)	63 (72%)	47 (89%)	21 (84%)	171 (81%)	*0*,*06*
IMC (≥30)	7 (15%)	13 (15%)	9 (17%)	7 (28%)	36 (17.1%)	*0*,*48*
Age (mean±SD)	66±8	71±9	66±9	74±12	69±10	*0*.*001*
eGFR^a^ <30ml/min	0 (0%)	4 (4.6%)	2 (3.8%)	2 (8%)	8 (3.8%)	*0*.*41*
eGFR <60ml/min	8 (17.4%)	28 (32.2%)	10 (18.9%)	12 (48%)	59 (27.5%)	*0*.*049*

^a^eGFR: estimated glomerular filtration rate

Sample collection and handling was performed in accordance with the guidelines of the medical and ethical committee in Nantes, France, and all patients participating in the study provided written informed consent (research protocol#PFS09-014, authorized on Dec 23, 2009 by the agence de biomédecine). In case of organ donors, the absence of patient opposition to organs donation and an informed and signed consent was required from the patient’s family. In all cases the priority was given to the therapeutic harvesting rather the scientific harvesting. Legal and ethical authorizations have been granted by the french research ministry (n°DC-2008-402), the national commission for computerized information and liberties (CNIL, n°1520735 v 0), and the local ethical committee (groupe nantais d'éthique dans le domaine de la santé, GNEDS). For carotid and femoral arteries, endarterectomies were performed on a consecutive series of patients using conventional surgical techniques. For carotid and femoral arteries, the sample was limited to one lesion since endarterectomies were performed. The plaque was removed at the bifurcation from the lumen as a single specimen. For aorta and infrapopliteal arteries, the sample was harvested with the most severe atheromatous lesion seen during the procedure, and surgeons collected a similarly sized sample as carotid and femoral specimens. All samples were 1-2cm long. For histology, we analyzed sections of the core of the lesion present in each arterial sample.

None atheromatous arteries were collected from arterial allograft donors. Sample collection and handling was performed in accordance with the guidelines of the medical and ethical committee in Nantes, France, and all patients or patient’s next of kin provided written informed consent.

Exclusion criteria for non-atheromatous arteries and patients were history of cardiovascular diseases (ischemia cardiopathy, stroke, peripheral artery diseases), or presence of macroscopic athero-thrombosis during tissue collection.

### Histological and immunological analyses

For histological analysis, the tissue samples were fixed in 10% formalin for 24-48h, decalcified in Sakura TDE 30 fluid for 5 days, and embedded in paraffin. Ten serial sections from the middle of the atherosclerotic lesion were processed. These sections were stained with hematoxylin and eosin (HE, Sigma Aldrich, Saint-Louis, MO, USA). Grading of lesion types was based on the updated AHA classification [18]. Immunohistochemistry (IHC) was performed to localize and semi-quantify endothelial cells with CD31 antibody (Dako, Glostrup, Denmark), pericytes with NG2 antibody (Millipore, Billerica, MA, USA), smooth muscle cells with smooth muscle actin-α (α-SMA, R&D Systems, Minneapolis, MN, USA), and macrophages with CD68 antibody (Immunotech, Marseille, France). For each IHC staining, a negative control excluded the primary antibody. Whole sections were imaged with the NanoZoomer digital slide scanner (Hamamatsu Photonics, Hamamatsu, Japan).

For each patient, the whole slide was scanned. We quantified positive stained area using Image Pro Plus (National Institutes of Health, Bethesda, MD, USA) with similar parameters for all samples. Numbers were expressed as a percentage of the stained area over the area of the plaque for each patient, and final results are the mean of the whole cohort. We analyzed the presence of calcification types (sheet like, nodular, clear center/micro calcifications, and osteoid metaplasia) by 2 independent investigators.

### Cell isolation, culture and differentiation assays

Fresh tissues are collected, stored in cold PBS for 3–6 hours before processing. We washed the sampled with cold PBS then dissociated non-atheromatous arteries from organ donors with an enzymatic mix including by collagenases (Liberase DL, Roche), elastase (porcine elastase, Sigma-Aldrich) and DNAse (DNAseI, Sigma-Aldrich) in High glucose DMEM. A first short digestion step permitted endothelial cell isolation. Then mechanical dissociation followed by a longer incubation with digestion mix allowed smooth muscle cells isolation.

We removed occlusive debris from cell suspension using 7μm mesh strainer. Additional centrifugation removed smaller debris and cells were plated in gelatin-coated (1% porcine type B gelatin, Sigma-Aldrich) 12 wells plate (Corning cell bind, Corning) in complete media ECBM2 and SMCBM2 for endothelial cell (EC) and SMC respectively (Promocell, Heidelberg, Germany). In case of EC/SMC mixed populations, we performed an immune-magnetic separation using CD31 beads (anti-human CD31 beads, Thermo Fischer scientific).

The SMCs used in the study were isolated from non-atheromatous arteries of various arterial location of the same organ donors. These medial derived SMCs were used between 3 and 5 passages. Exclusion criteria for non-atheromatous arteries group were history of cardiovascular diseases (ischemia cardiopathy, stroke, peripheral artery diseases), or presence of atherosclerotic lesion during tissue collection. We did not observe medial calcification for these samples by histology, as the sample was divided in separate pieces to perform such analysis.

We repeated the experiments for 5 independent donors (3 males, 2 females), from 18 to 64-year-old (mean 45.8+/-7.9). There was no significant difference in age between groups.

SMC were differentiated into osteoblastic cells by adding 1.8–3 mM inorganic phosphate to 3% Fetal Calf Serum (FCS) containing DMEM culture medium, as previously described [19]. SMC differentiation and mineralization was assessed 7–9 days after treatment, using alizarin red staining (Sigma-Aldrich).

Assessment of TGFβ Receptor 1 (TGFβR1) signaling activity was evaluated through the levels of phosphorylated smad3. Induction consisted in treating cells with TGFβ for 45 minutes. SD208 (2-(5-Chloro-2-fluorophenyl)pteridin-4-yl]pyridin-4-yl-amine, Sigma-Aldrich) was added 1h before TGF treatment. Antibodies for P-smad3 and Smad3 were purchased from Millipore, and vinculin from Cell Signaling Technology.

### Gene expression and microarray analysis

Total RNA from SMC was extracted using TRIzol reagent (Invitrogen Life Technologies, Inc.). For microarray analysis, RNA was hybridized to Agilent human gene expression microarrays (G4851C). Fluorescence values corresponding to raw expression data were extracted. Positive and negative control probes were removed. Non-linear effects such as background or saturation were corrected by Lowess [20] against a median profile of all samples [21]. Values of replicate probes were averaged and the data matrix was filtered to 20,000 probes based on highest median expression values. Clusters of co-expressed genes were identified using K-means (k = 11) on natural-log-transformed and gene-median-centered data with uncentered correlation as a similarity metric in Gene Cluster 3.0 [22]. The value of k was determined by analyzing the presence of novel phenotype-specific gene clusters after incrementing k (start value k = 10). Hierarchical clustering was performed using Gene Cluster 3.0 and heatmaps were displayed using Java Treeview. Clusters separating the different arterial beds were selected and a collective p-value was calculated. For each sample, a mean expression value of all genes was calculated. The mean values of different arterial beds were compared. This strategy based on strong correlation of gene expression allowed us to avoid multitesting. Hierarchical clustering of these clusters demonstrated a great homogeneity.

Microarray data can be found at: http://www.ncbi.nlm.nih.gov/geo/query/acc.cgi?token=ibwbossexboxzmt&acc=GSE84012

Gene Ontology enrichment analysis of the different clusters was performed using GoMiner. [23] Enrichment of GO terms were determined using the 20,000 probes list as background. Annotations with FDR<0.05 were considered significant.

For specific gene quantification, total RNAs were also reversed transcribed using the transcriptor first strand cDNA synthesis kit (Roche Applied Science). Real-time monitoring of PCR amplification of complementary DNA (cDNA) was performed using DNA primers on CFX96 detector system (Bio-Rad) with SYBR PCR Master Mix (Bio-Rad). Target gene expression was normalized to *HPRT* levels in respective samples as an internal standard, and the comparative cycle threshold (Ct) method was used to calculated relative quantification of target mRNAs: *runt-related transcription factor 2* (*RUNX2*) (F-GTGCCTAGGCGCATTTCA/ R-GGCTCTTCTTACTGAGAGTGGAAG), *osteocalcin* (*OC*) (F-GGCGCTACCTGTATCAATGG/ R-TCAGCCAACTCGTCACAGTC), *alkaline phosphatase* (*ALP*) (F-AACACCACCCAGGGGAAC/R-GGTCACAATGCCCACAGATT), *TGFΒR1* (F-GCAGACTTAGGACTGGCAGTAAG/R-AGAACTTCAGGGGCCATGT), *SMAD7* (F-TTTGCCTCGGACAGCTCAAT/R-TTTTTGCTCCGCACCTTCTG), *SERPINE1* (F-GCTTTTGTGTGCCTGGTAGAAA/R-TGGCAGGCAGTACAAGAGTGA), *HPRT* (F-TGACCTTGATTTATTTTGCATACC/R-CGAGCAAGACGTTCAGTCCT).

### Statistics

Variables are displayed as mean ± SD. Two investigators performed the morphologic observations independently and blindly, without any indication on the arterial bed or patient group. Data were analyzed by the Kruskal–Wallis one-way analysis of variance and Bonferroni post-test for each factor. Population statistics between ECLAGEN groups was analyzed by Fisher’s exact test and global ANOVA for age comparison. Statistical analyses were performed using Graphpad Prism (GraphPad Software, Inc., La Jolla, CA, USA). A p-value < .05 was considered statistically significant. All in vitro experiments were performed all least 3 times, with 3 independent samples preparations.

## Results

### Atherosclerosis heterogeneity among vascular beds

**Fig 1:**
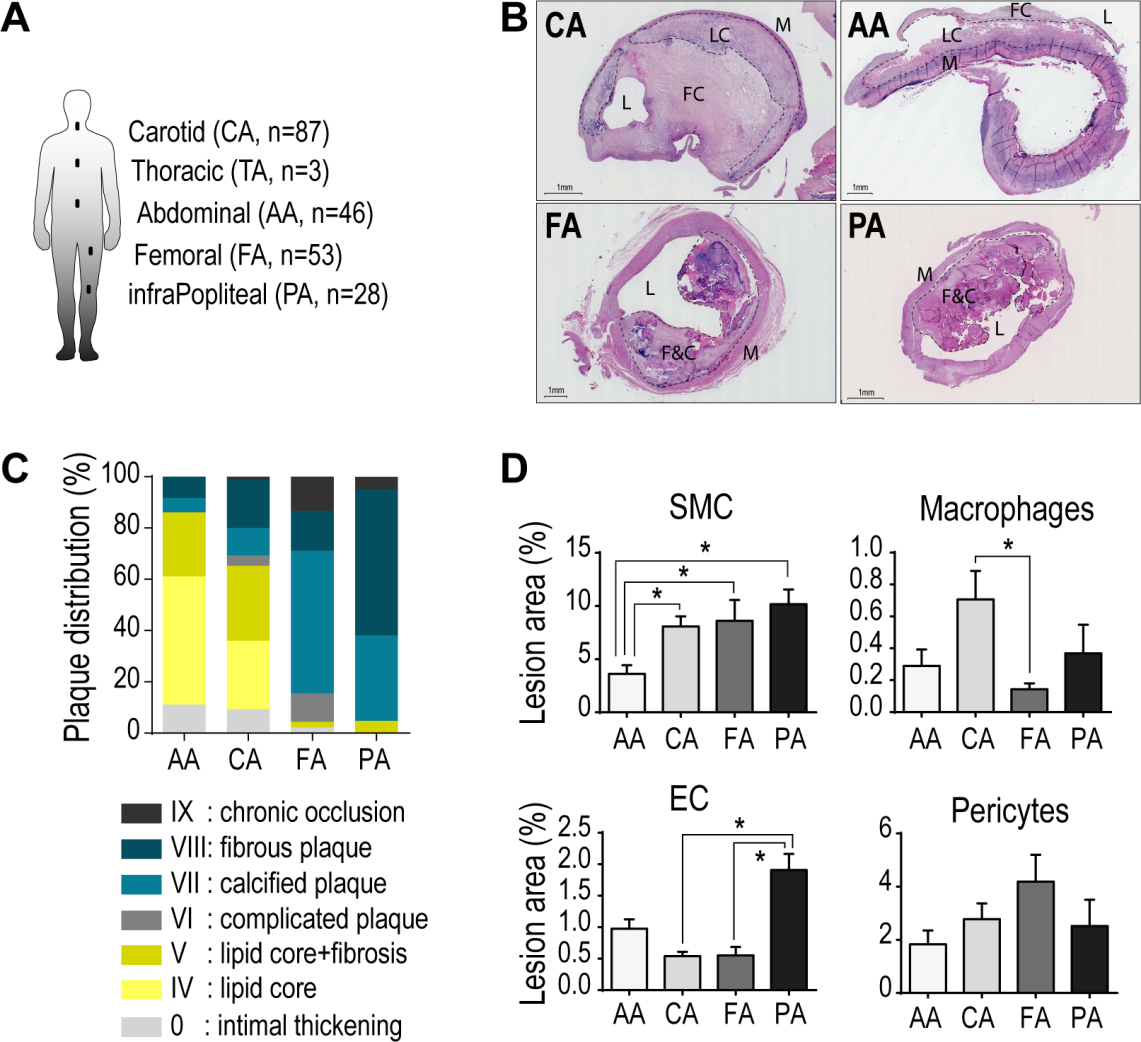
Heterogeneous atherosclerotic lesions among arterial beds. (A) Atherosclerotic lesions from patients (P) and healthy arteries (H) were collected. (B-C) Representative images of hematoxylin/eosin (HE) stained lesions and AHA classification of plaque composition from each arterial location. (D) Quantification of immunohistochemical stainings for endothelial cell, SMC, macrophage, and pericyte in lesions from each arterial bed. Bars represent mean ± SD (*p<0.*05*). Lumen (L), Media (M), Lipid core (LC), Fibrous Cap (FC), and Fibrosis and Calcification (F&C).

We previously reported a drastic difference in plaque composition between carotid and femoral arteries with our first human lesion biocollection ECLA [8,9,11,24]. ECLAGEN biocollection comprises 230 patients. We collected a total of 308 lesions and healthy arteries from abdominal (AA, n = 46/11, patient/healthy) and thoracic aorta (TA, n = 3/19), carotid (CA, n = 87/20), femoral (FA, n = 53/20), and infrapopliteal arteries (PA, n = 28/21) (Fig 1A). Healthy arteries were obtained from healthy organ donors, and multiple locations were collected from the same organ donor for a more reliable comparison (*in vitro* work). The histological characterization of plaque morphometry and composition confirmed specificities in plaque development depending on anatomical location (Fig 1B).

Abdominal and carotid arteries develop predominantly lipid-rich lesions, with a marked presence of lipid core (75% and 56%). Femoral and infrapopliteal arteries exhibit highly fibrotic and/or calcified lesions. Plaques from infrapoliteal arteries are largely fibrotic (57%), but also develop large calcification (33%). Femoral arteries present mostly calcified lesions (56%) and fibrotic lesions (16%). Only scarce lesions with lipid inclusions were found in femoral and infrapopliteal arteries (Fig 1C).

Cellular composition reflected plaque diversity between typical atheromatous carotid lesions, enriched in lipids and with highest macrophage content among vascular beds, and lower limb arteries that develop stenotic lesions with extensive fibrosis, with scarce macrophage presence. Femoral arteries and infrapopliteal arteries are both associated with increased neoangiogenesis, reflected by a significant increased presence of endothelial cells and pericytes within the diseased intima (Fig 1D).

### Plaque heterogeneity among vascular beds correlates with differences in calcification burden

**Fig 2:**
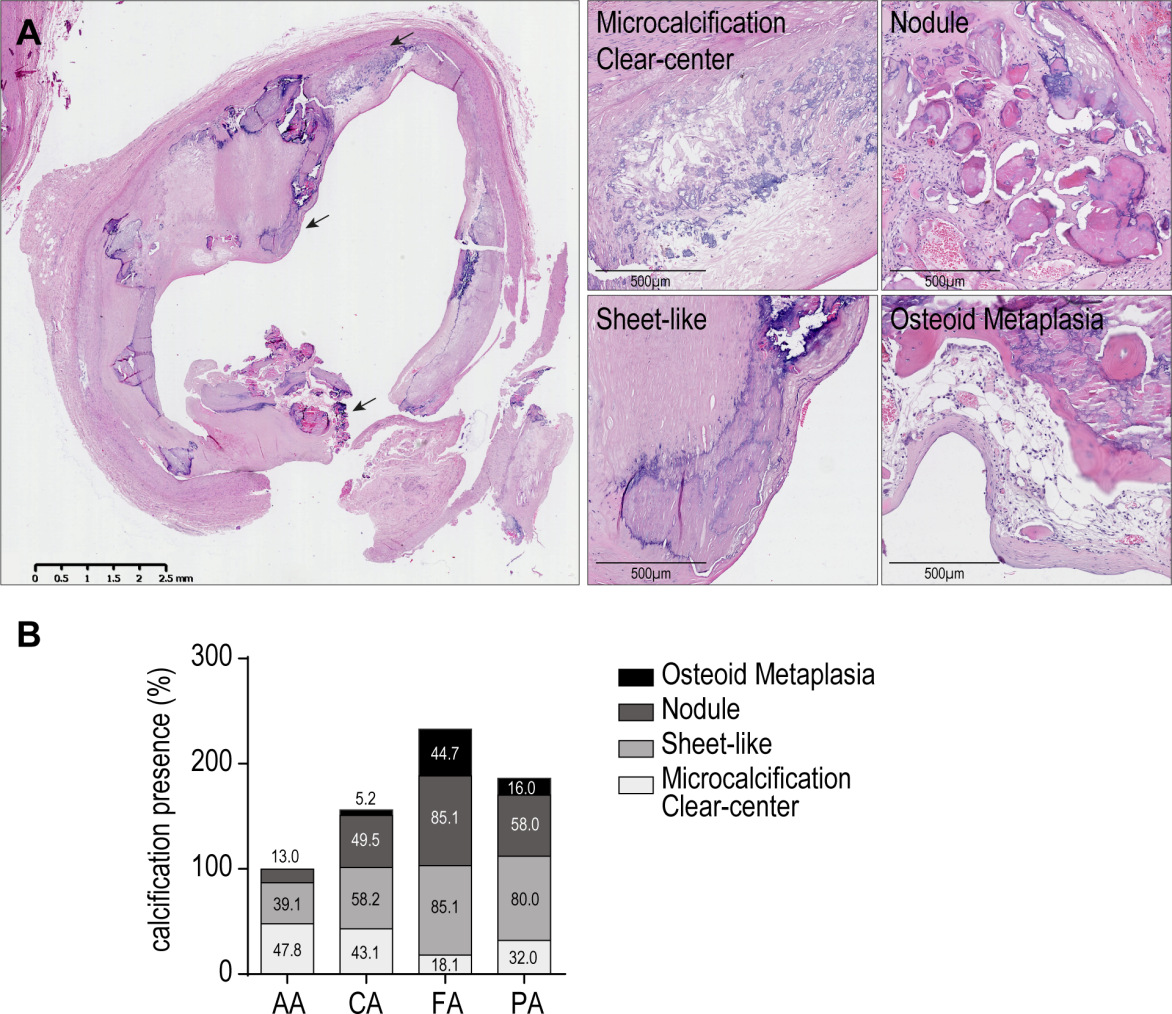
Heterogeneous calcification types among arterial beds included in ECLAGEN biocollection. Atherosclerotic lesions often present multiple calcification types (arrows). (A) Microcalcification/clear center calcification, sheet-like calcification, calcified nodule, osteoid metaplasia. (B) Differential presence of calcification types among lesions from each arterial location. (AA, n = 46; CA, n = 87; FA, n = 53; PA, n = 28).

Vascular calcification is a general term that encompasses various forms of mineralized structures. We and others previously identified four main types of intimal calcification in atherosclerotic lesions [9,25]. Clear center/microcalcification–often present in close proximity from lipid-rich core, sheet-like calcification–stratified areas of mineralizing tissue, nodules, and osteoid metaplasia–actual bone structure characterized by the presence of osteoid tissue with the presence of osteocyte-like cells, multinucleated osteoclast-like cells, and a lipid and leucocytes-rich pseudo bone-marrow (Fig 2A) [11,26].

Only few samples in this cohort were found without any form of calcification (18%). The abdominal aortas present the least calcification burden overall, with mostly lipid-related microcalcification (present in 47.8% of all aortic lesions) and sheet-like calcification (39.1%). Infrapopliteal arteries and femoral are more prone to develop dense and structured calcification compared to aorta and carotid arteries (sheet-like: 85.1% (FA) and 80.0% (PA) vs 39.1% (AA) and 58.2% (CA), and nodules: 85.1% (FA) and 58.0% (PA) vs 13.0% (AA) and 49.5% (CA)).

Femoral arteries are significantly more calcified than any other vascular bed, with a high prevalence of all advanced calcification types, and specifically OM (44.7%), far more frequent in femoral than in carotid (5.2%), aorta (0%) or infrapopliteal arteries (16.0%). This calcification heterogeneity, together with plaque composition, suggests that specific local environment or/and molecular determinants orient plaque development and calcification formation depending on the anatomical location.

To assess whether predominant atheromatous cell types (macrophage, smooth muscle cell, endothelial cell, and pericyte) could play a direct role in each calcification types formation and evolution, we evaluated their quantitative and qualitative association (S1 Fig).

**Fig 3:**
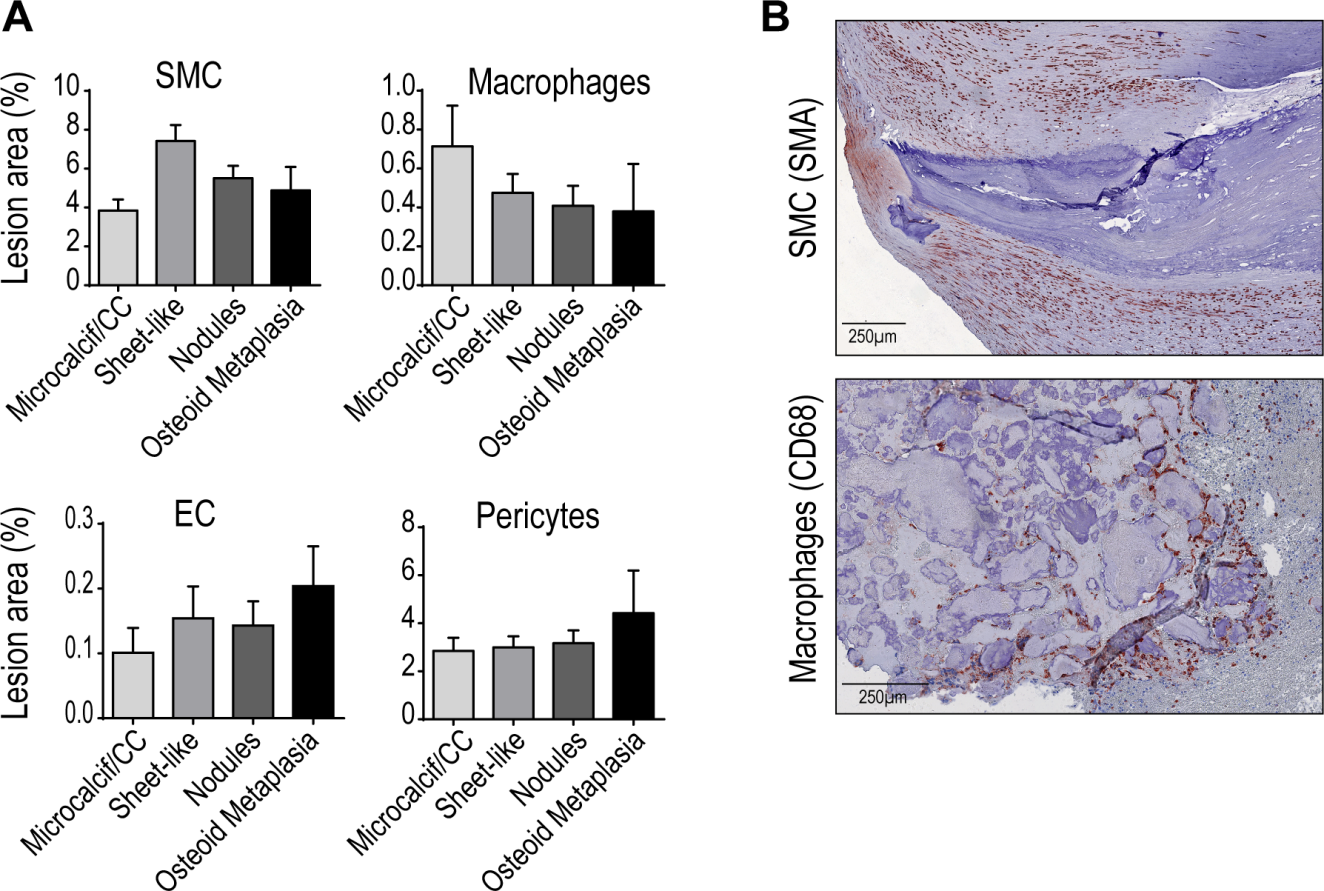
Cell content associated with vascular calcifications. (A) Quantification of endothelial cell, SMC, macrophage, and pericyte content in lesions with given calcification types (A) (AA, n = 46; CA, n = 87; FA, n = 53; PA, n = 28). (B) Representative images illustrating the close proximity of SMC and macrophages with sheet-like and nodular calcifications, respectively. Bars represent mean ± SEM.

Quantitative analysis for each staining did not reach any significant difference in SMC, macrophage, EC and pericytes with any calcification type (Fig 3A). Many of the lesions analyzed presented several types of calcification, which makes the quantitative analysis difficult to interpret at the whole lesion level. We observed a trend towards a quantitative association between SMC with sheet-like, and EC/pericytes with OM, as in our previous study [8]. We better estimated the link between cell and calcification types by analyzing their spatial proximity. Sheet-like predominantly develop and emerge from SMC-rich fibrotic parts of the lesions, while macrophages are often found in close proximity or direct contact with calcified nodules, suggesting a direct implication of these cell types in their respective formation (Fig 3B).

### SMC from none atheromatous femoral arteries are more prone to mineralization

**Fig 4:**
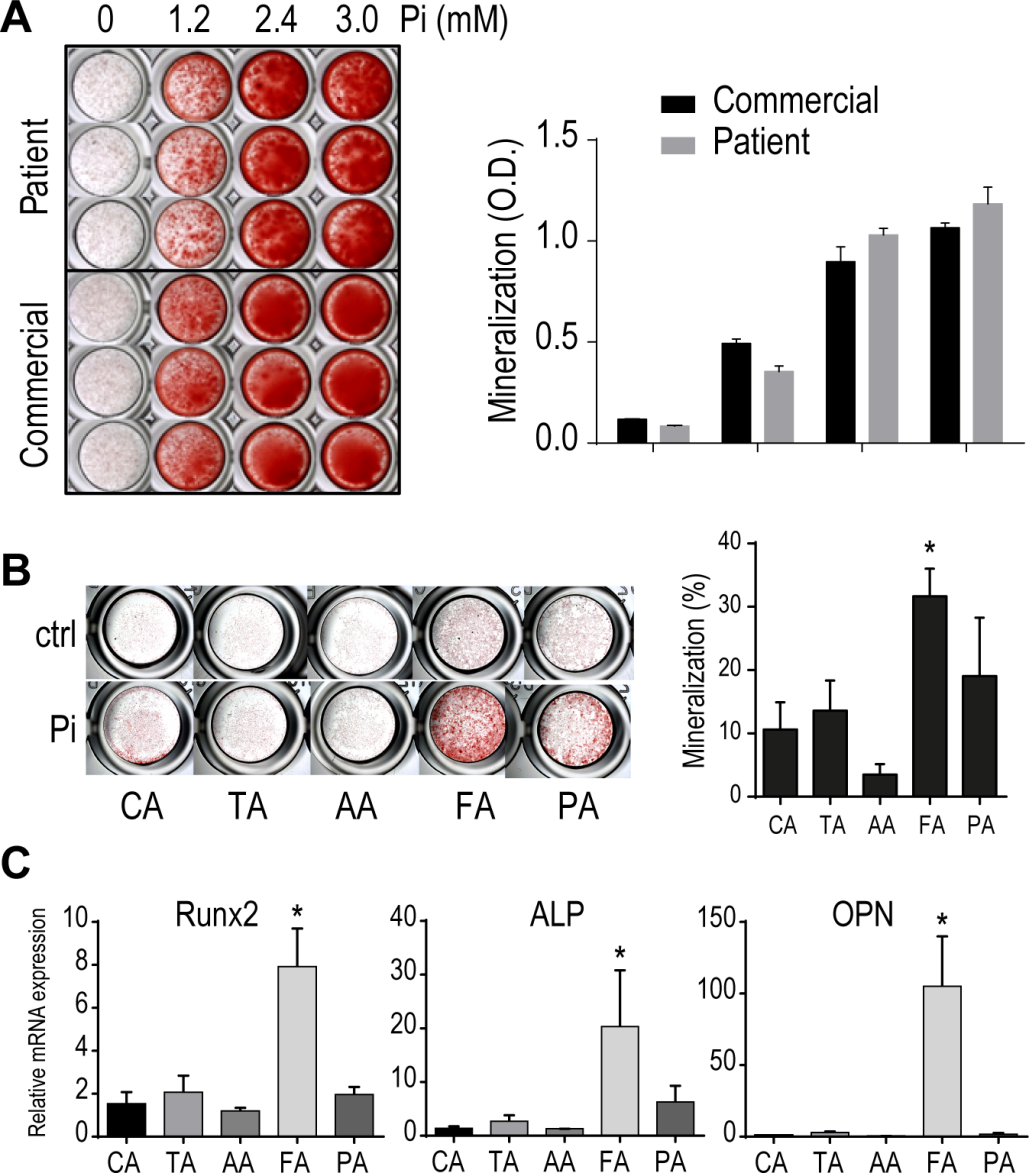
Differential mineralization properties of SMC depending on their anatomical origin. (A) Mineralization of commercial and SMC isolated from healthy thoracic aorta (ECLAGEN sample) 10 days after treatment with indicated concentrations of inorganic phosphate. (B) Mineralization after 7 days of SMCs isolated from each healthy anatomical location, in presence of 2.4 mM inorganic phosphate (mineralized area in %). (C) Differential expression of osteoblastic markers Runx2, ALP and OPN in SMCs from different arterial beds. Bars represent mean ± SD. (*p<0.05).

Among atheromatous cells, SMCs are a prominent active player in vascular calcification due to their mineralizing properties when subjected to a pro-osteogenic milieu. The close localization of SMCs to sheet-like calcifications in plaques and to medial calcification in chronic kidney disease (CKD) patients further advocates for a direct role of SMC in plaque mineralization. To assess the contribution of SMC in vascular calcification heterogeneity among vascular beds, we isolated primary medial SMC from healthy abdominal and thoracic aorta, carotid, femoral and infrapopliteal arteries from given non-atherosclerotic organ donors. We induced cell mineralization in presence of 1.2–3.0mM inorganic phosphate in culture medium for 7 days. Our own primary SMCs showed similar mineralizing capacities as commercial SMC, with no difference in cell viability (Fig 4A).

We cultivated SMC from the 5 healthy arterial locations for 7 days in osteogenic condition. The results shown in Fig 4B indicate that SMC from femoral arteries mineralize 39.8% more than infrapopliteal arteries, and than other vascular beds (66.5% vs CA, 57.0% vs TA, and 88.9% vs AA). Differential ability to mineralize was also reflected by a marked increase in osteoblastic marker expression (runx2, alkaline phosphatase, osteopontin) in femoral SMC compared to other arterial beds (Fig 4C). SMC mineralization capacities correlated with the extent of plaque calcification observed in patient lesions, suggesting that specific propensities among SMC to mineralize could directly contribute to atheromatous calcification heterogeneity in peripheral arteries.

### SMC from peripheral arteries express specific transcriptomic signatures

Vascular calcification heterogeneity among vascular beds could either derive from local microenvironmental specificities and or reflect molecular singularities between arterial beds. To analyze these differences and identify key molecules involved in this process, we performed a transcriptomic microarray analysis with medial SMCs isolated from the 5 healthy peripheral arterial beds indicated above (from 54 and 64-year-old organ donors) (S1 File).

**Fig 5:**
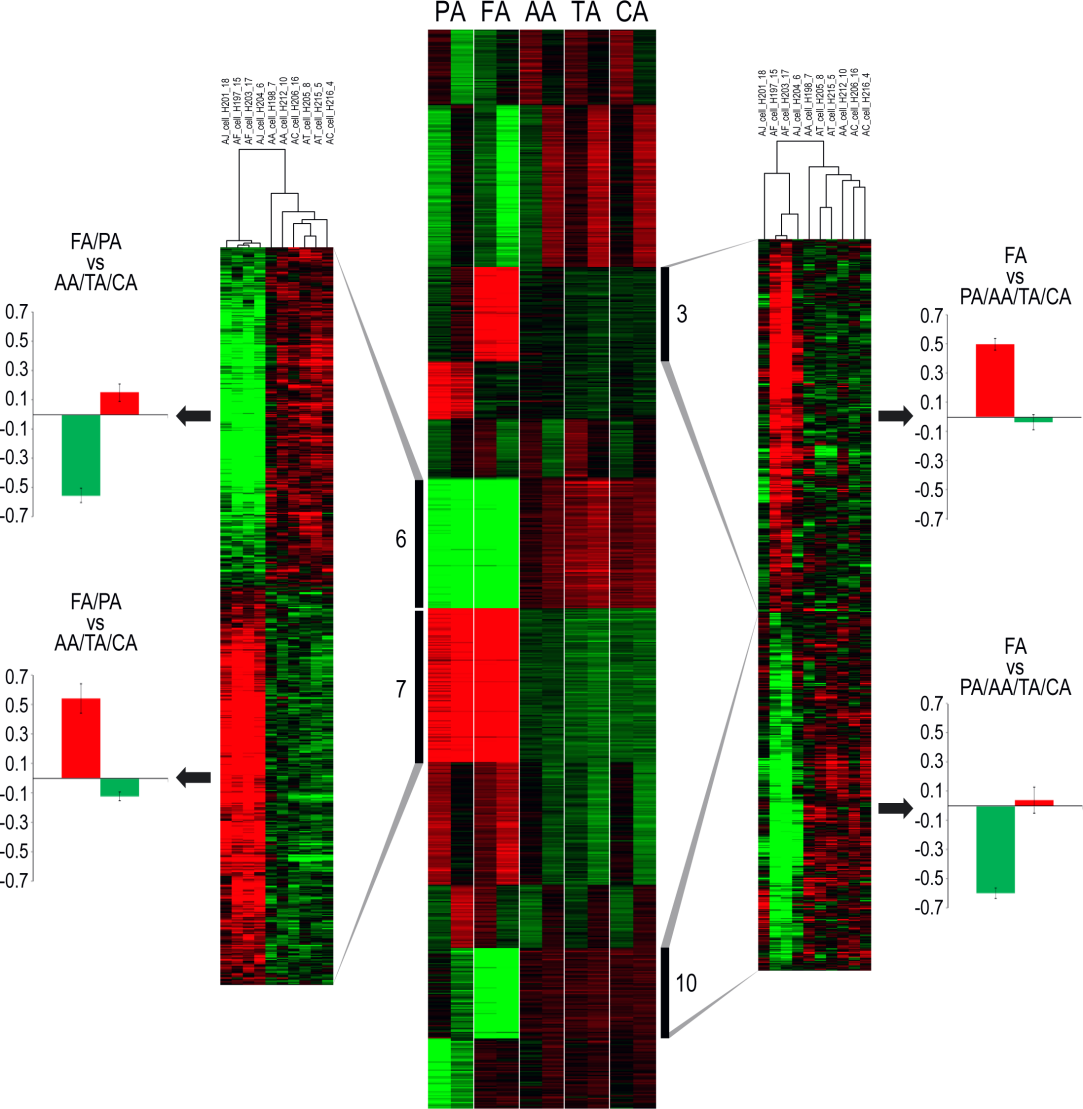
Transcriptomic heterogeneity of SMCs originating from distinct arterial locations. Gene expression is presented as a colored matrix where each row represents a gene and each column a sample. Green, black and red correspond to lower values, median values and higher values, respectively. Middle: Transcriptome data clustered by K-means (with k = 11). Clusters 6 and 7 (hierarchical clustering shown on the left) clearly separate FA/PA and AA/TA/CA arterial beds. Clusters 3 and 10 (hierarchical clustering shown on the right) display an FA-specific gene expression profile. Extreme left and right: For each sample, the average of all probes was calculated.

K-means analysis of the microarray data identified several gene clusters with arterial bed(s) specific expression profiles (Fig 5).

Incrementing k from 10 to 11 revealed the presence of an FA-specific gene cluster (cluster 10). Further increasing k to 12 or 13 did not reveal any novel phenotype-specific gene clusters. Therefore, k was fixed at 11. Two clusters were selected because of striking differential expression levels between FA/PA and AA/TA/CA arterial beds (cluster 6 (2437 probes) and cluster 7 (2846 probes)). Two additional clusters were selected because of an FA-specific gene expression profile (cluster 3 (1748 probes) and cluster 10 (1679 probes)). Functional annotation analysis showed that genes downregulated in FA/PA vs. AA/TA/CA arterial beds (cluster 6) were mostly involved in endocrine system development, translational termination, macromolecular complex disassembly and antigen processing and presentation. Enriched annotations among the upregulated genes (cluster 7) included functions related to intracellular transport and protein localization. Genes highly expressed in the FA arterial bed only (cluster 3) were notably involved in cellular carbohydrate metabolic processes. Genes downregulated in the FA arterial bed vs. all others (cluster 10) were involved in the immune response. This gene cluster also contained genes involved in ossification and lipid localization.

As femoral arteries are more prone to mineralize, we screened for important signaling pathways linked to osteoblastic differentiation and tissue mineralization in cluster 3 and 10. Among the genes specifically overexpressed or absent in FA compared to other vascular beds, we found that 19 members of TGFβ signaling.

This suggests that TGFβ signaling might play a role in the differential mineralization capacities of SMC depending on their anatomical origin.

### Differential TGFβ signaling in SMC from different arterial beds accounts for SMC mineralization heterogeneity

**Fig 6:**
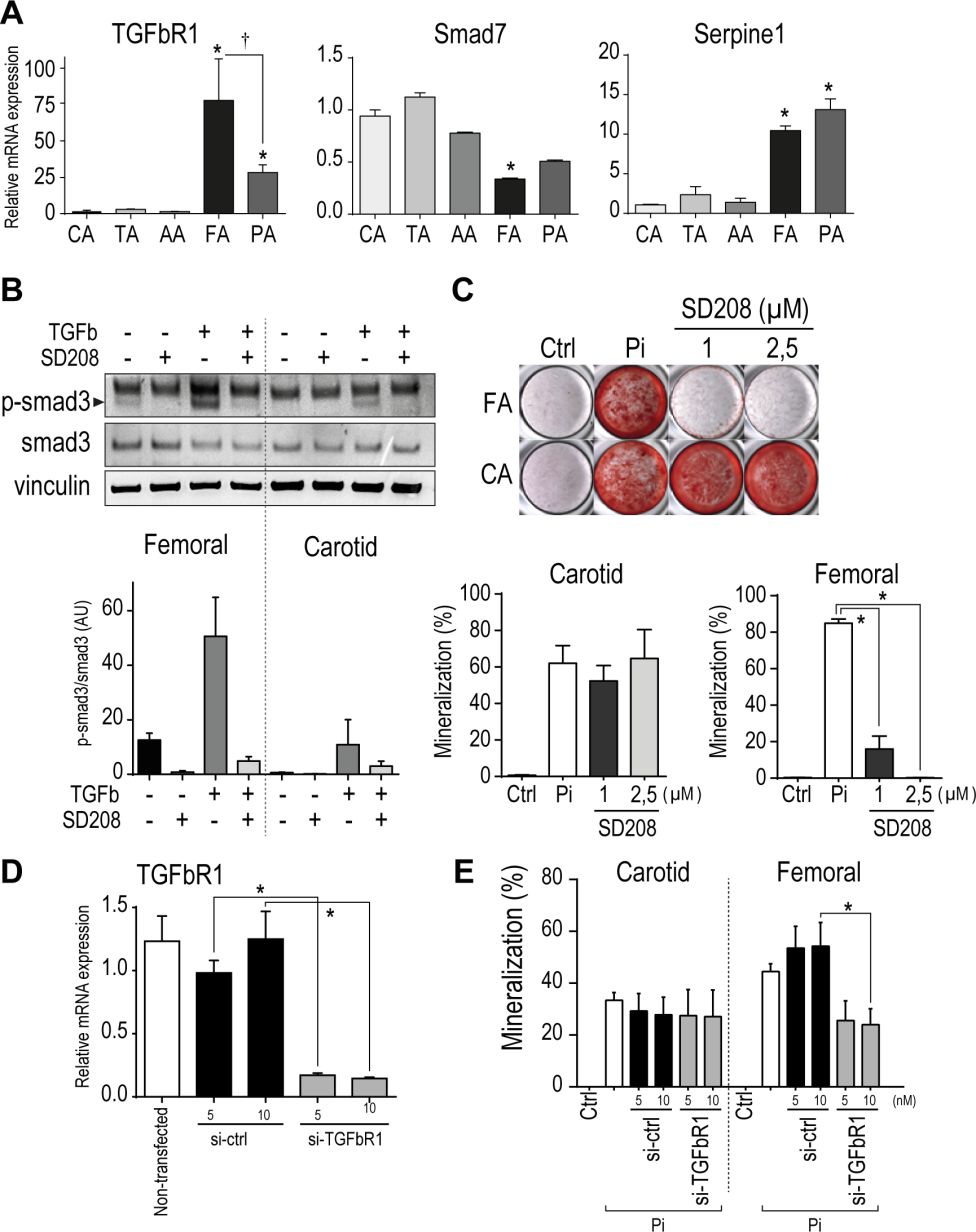
Functional implication of TGFβ signaling in SMC mineralization heterogeneity. (A) Relative mRNA expression level of TGFβR1, Smad7 and Serpine1 in resting SMCs from each arterial bed. (B) Basal and induced phosphorylation of TGFβR1 effector protein smad3 in presence or absence of TGFβR1 inhibitor SD208 (1μM). (C) Mineralization of SMCs from carotid and femoral arteries in presence of inorganic Phosphate (2.4mM) and SD208 for 9 days. (D) TGFbR1 expression knockdown in SMC 48hrs post-transfection. (E) Mineralization of SMCs from carotid and femoral arteries after TGFbR1 knockdown in presence of inorganic Phosphate (2.4mM) 7 days. Bars represent mean ± SEM. (*p<0.05).

Gene screening from transcriptomic analysis led to the identification of TGFβ signaling, specifically upregulated in femoral arteries. Individual qPCR analysis confirmed elevated levels of TGFβR1 in femoral SMC, compared to infrapopliteal arteries (2.7 fold), carotid (70.5 fold), and aorta (49 fold). Additional TGFβ signaling related molecules (smad7, serpine, bambi) have been found differentially expressed in femoral and infrapopliteal arteries compared to other arterial beds (Fig 6A).

We confirmed higher basal and induced TGFβR1 signaling in femoral-derived SMC compared to carotid-derived cells, through the increased presence of phosphorylated smad3 (Fig 6B).

Because TGFβ has been already reported in cell mineralization process and vascular cell transdifferentiation in osteoblast-like cells [27–29], we postulated that high propensity of femoral cells to mineralize could derive from an increase presence of TGFβ signaling. To assess this question, we treated femoral and carotid arterial SMC with TGFβ signaling inhibitor SD208 while inducing osteobastic differentiation with high inorganic phosphate medium. Fig 6C and 6E show that TGFβ blockade with SD208 at 1 and 2.5μM or targeted siRNA was sufficient to efficiently prevent femoral cells mineralization, but not for carotid SMC, confirming a specific role of TGFβ signaling in SMC mineralization in an anatomical dependent manner.

## Discussion

Our work further explores the differential development of atherosclerosis among vascular beds and suggests potential clinical implications. A better understanding of the mechanisms regulating plaque development and evolution towards a typical lipid-rich inflamed vulnerable lesion, a fibrous, or a highly calcified plaque could lead to new therapeutic strategies for preventing acute athero-thrombotic events.

We and others have previously reported that carotid and femoral arteries develop very different lesion types [9,30]. While exposed to the same cardiovascular risks, carotid arteries predominantly present classical atheromatous lesions, and femoral arteries develop fibro-calcic lesions. Our present study, that includes additional vascular beds, confirmed and extended this plaque heterogeneity characterization. Infrapopliteal arteries, anatomically close to the femoral bed, show similarly fibrotic and calcified lesions. Sheet-like calcifications (found in the media of chronic kidney diseases) are the predominant form of plaque mineralization in infrapopliteal arteries, with limited presence of microcalcifications, nodules and bone tissue, the latter being found almost exclusively in femoral arteries. Carotid lesions develop all kinds of calcification, compared to the aorta that seems overall less prone to mineralize.

Our observations strongly suggest that microcalcification derive from the lipid-rich, necrotic core in inflamed lesions, supporting a role of apoptosis and necrosis in early calcification events, as previously reported [31,32]. The more structured calcifications do not quantitatively correlate with SMC, macrophages or EC content within the lesion, but qualitative analysis indicates that sheet-like calcifications develop in close proximity of SMC-rich areas, and macrophages often associate with calcified nodules. This observation further supports the contributing role of SMC and macrophages in vascular calcification. SMCs are believed to play an active role in plaque mineralization through their release of calcifying matrix vesicles and their osteogenic differentiation ability [33–35]. Macrophages have also been reported to play a supportive role in plaque mineralization through the release of matrix vesicles and by their lineage with osteoclastic cells [36–38]. A more thorough histological with multiple co-stainings would attest the real phenotype of macrophages and smooth muscle cells present in each lesion, as CD68 and SMA can be also expressed at lesser levels by other myeloid and mesenchymal-derived cell types, respectively, and with respect with recent work suggesting that SMC could even transdifferentiate into macrophage-like cells [39].

The quantitative analysis failed to illustrate these associations, because most of the lesions exhibit several types of calcification. The discrepancies among calcification types (localization, morphology, associated lesions and territories) strongly suggest that specific mechanisms may regulate their formation and development. Direct and systematic chronological link between microcalcification, sheet-like, nodule and OM seems unlikely, based on the drastic difference in associated plaque composition. This critical question remains unexplored due to the lack of technological imaging modality capable of detecting and following up each calcification form over time in given lesions.

One limitation of such study also remains that diseased specimens from each vascular bed was obtained from different patients, and other vascular beds cannot be analyzed besides those operated upon.

Calcification and plaque composition heterogeneity among vascular beds could derive from specific local microenvironmental (e.g. hemodynamics), intrinsic biological singularities that orient and regulate plaque evolution and mineralization, or both. Clinical data analysis did not show any significant impact of common cardiovascular risk factors (age, sex, diabetes, dyslipidemia) with calcification, all types included, nor with any given calcification type. Sulkava *et al* recently confirmed plaque heterogeneity among vascular beds at transcriptomic levels [40]. Our results show SMC heterogeneity among vascular beds, with marked specific transcriptomic signatures between lower (femoral and infrapopliteal arteries arteries), and upper body (aorta and carotid arteries), even several months of culture following isolation from healthy arteries. Our results provide molecular evidence that SMC differ between arterial beds. The differential expression of numerous developmental genes in our transcriptomic analysis may reflect their different embryonic origins [41,42], and is reflected by SMC heterogeneity [43].

TGFβ signaling plays a critical role in atherosclerosis, with important contribution many aspects of the disease (hyperlipidemia, hypertension, immunomodulation, thrombosis, and vascular remodeling). TGFβ is in particular a key regulator of SMC phenotypic switch leading to SMC migration, proliferation, matrix production, but also mineralization [29,44,45]. Our transcriptomic analysis indicates a marked difference in basal TGFβ signaling or TGFβ sensitivity in arterial beds. Overexpression of TGFβR1 in femoral arteries is likely to favor SMC switch, mineralization, and hence calcification. In addition to specific local microenvironment, the marked vascular cell (SMC, EC) heterogeneity demonstrated by our transcriptomic analysis and other reports, is a key element to better understand plaque biology and development.

## Supporting information

S1 Fig Plaque Heterogeneity among vascular beds. Representative images of Hematoxylin/Eosin (HE) and Masson’s Trichrome colorations, and immuno-histochemical staining for endothelial cell (CD31, brown), SMC (α-smooth muscle actin, red), and macrophage (CD68, brown). Lumen (L), Media (M), Lipid core (LC), Fibrous Cap (FC), and Fibrosis and Calcification (F&C). Quantitative analysis was performed within the lesion as indicated by the dotted line.
(TIF)

S1 File Microarray analysis between SMCs isolated from healthy arterial beds. Values of replicate probes for 20k genes based on highest median expression values (Significance analysis of microarrays (SAM) analysis).
(XLSX)
