# Supplementary figures and images for: Implication of molecular vascular smooth muscle cell heterogeneity among arterial beds in arterial calcification
Source: PLoS One. 2018 Jan 26;13(1):e0191976. doi: 10.1371/journal.pone.0191976 (PMC5786328; doi:10.1371/journal.pone.0191976)

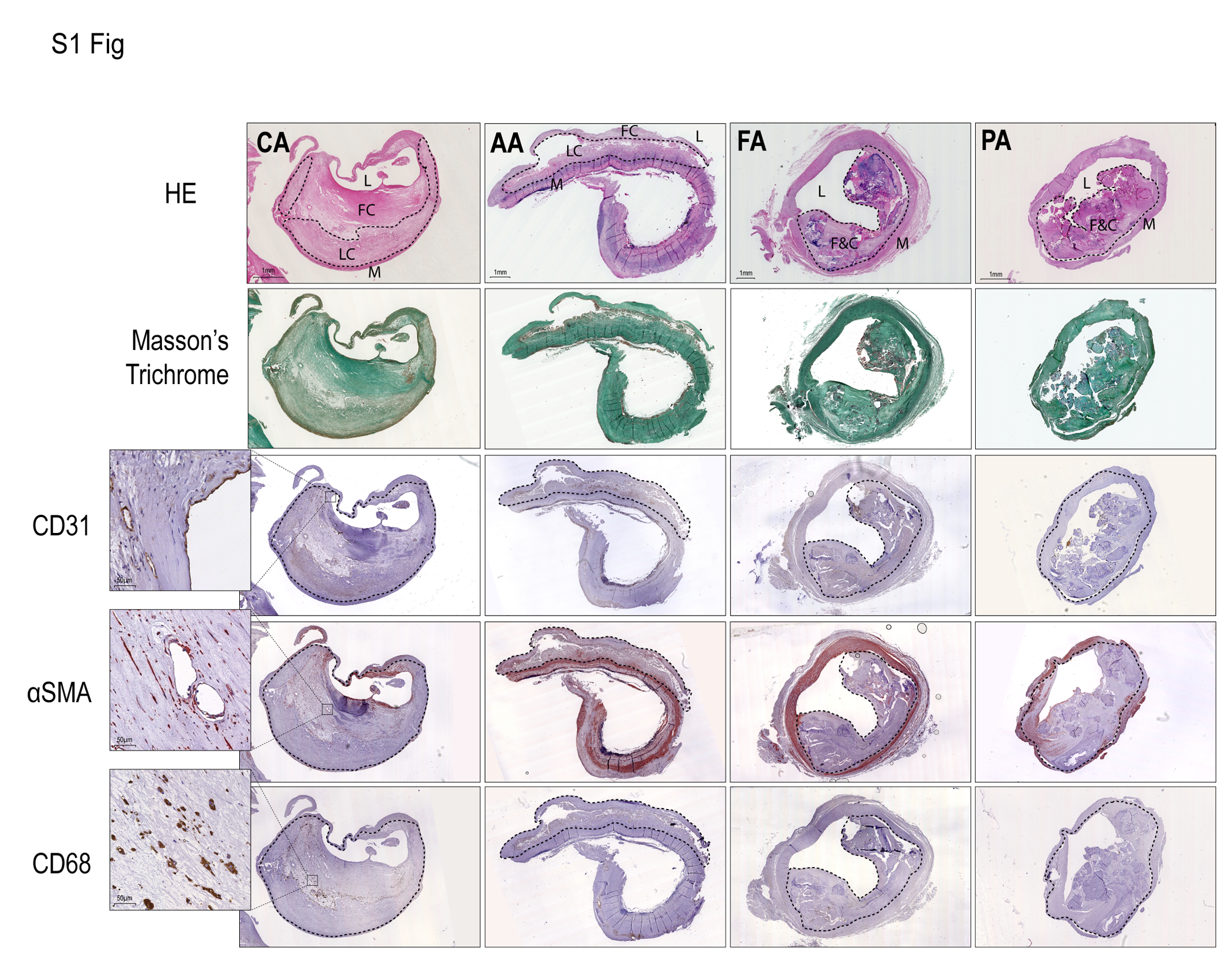

Supplement: S1 Fig — Representative images of Hematoxylin/Eosin (HE) and Masson’s Trichrome colorations, and immuno-histochemical staining for endothelial cell (CD31, brown), SMC (α-smooth muscle actin, red), and macrophage (CD68, brown). Lumen (L), Media (M), Lipid core (LC), Fibrous Cap (FC), and Fibrosis and Calcification (F&C). Quantitative analysis was performed within the lesion as indicated by the dotted line. (TIF) [file pone.0191976.s001.tif]
